# Supplementary material for: Access to and challenges in water, sanitation, and hygiene in healthcare facilities during the early phase of the COVID-19 pandemic in Ethiopia: A mixed-methods evaluation
Source: PLoS One. 2022 May 13;17(5):e0268272. doi: 10.1371/journal.pone.0268272 (PMC9106162; doi:10.1371/journal.pone.0268272)
Supplement: S1 File — (DOCX) [file pone.0268272.s001.docx]

**Annex I English version questionnaires**

The primary purpose of the inquiry is to improve the quality of water sanitation and hygiene in health facilities in South Wollo Zone. The effectiveness of the study will depend on the information you provide. The answers to the questions are for the purpose of the study.

**Thank you so much for your volunteering.**

**PART ONE GENERAL HEALTH INFORMATION**

1.1 Questionnaire Fill Date -----------------------------

1.2 District ---------------- City --------------------

1.3. Establishment of the institution ------------------------

1.4 Owner of Health Facility a) Private b) Government

1.5. Location of the Health institution A / urban b/ rural

1.6. Type of Health institution a) Clinic b) Hospital c) Health center d) other

1.7 Number of Employees M -------- F --------- Total------------------

1.8. Average number of patients per day --------------------

1.9. Is their WASH coordinator? a) Yes b) no

1.10. Is their WASH committee? a) yes b) no

**Part II. Water supply**

2.1. What is the main source of water in your institution?

A. tap water B/Protected dug well C / rain water D) Protected dug well

E/Tanker truck F/Surface water (River/ Lake/Canal) G/Tube well/ Borehole

H/ protected spring I/ un protected spring J/ others mention

2.2. Is their alternative water storage container? A / Yes B /no

2.3. Is the main water source is from the yard of your institution? A / Yes B /no

2.4 How far it is if you are outside of the yard A) Less than 500 m B) over 500 m

2.5. How long does it take to fetch water (on average minutes?)

A) Less than 5 B) 5-10 C) 10-15 D) Over 15

2.6. Is there a water supply right now A/ yes B/ no

2.7. Over the last couple of weeks, is there an entire supply of drinking water in the HCF

A / yes B) no

2.8. Is there Water supply for the year basis?

A) Yes all year round b) most of the time c) most of the time no supply

2. 8 Is their water quality monitoring program? A / yes B) no

2. 9 If the water type is a pipe, how many taps are available ------------

2.0 How many taps are currently in service ------------?

**Part Three sanitation**

3.1. Is there is a latrine in the health facility a) Yes b) no

3.2 If the answer is yes, the type of toilet in the health facility

A. Flush/Pour-flush to tank or pit B) Pit latrine without slab/open C) Pit latrine with slab/covered D) VIP E) mention if others

3.3 How many toilets you have in your intuition; -----------------

3.4 How many of them have lock and door ----------------------

3.5 Does the facility has separate toilets for clients and healthcare workers (A) Yes B) no

3.6. Does the facility have separate toilets for male and females? A) yes B) no

3.7 How often the toilet is cleaned

A) Twice a day B) Once a week C) Once a day D) specify any other e at any time

3.8 What does a sanitary toilet look like? A) High b) Medium c) Low

3.9 Is there a toilet suitable for people who are disabling? A) Yes B) no

3.10. What is the solution to be taken when the toilet is filled?

A) suck b) cover with soil c) None D / Explain if any other option

**Part four Hygiene facilities**

4.1 Is their functional handwashing sinks A) Yes B) no

4.2 number of latrines with only water --------------------

4.3 number of latrines with water and soap /ABHR---------------------

4.4. Is there a hand washing facility at point of care? a yes b/ no

4.5. Presence of handwashing facility (soap and water ABHR) at point of care and toilet

4.6. Presence of handwashing poster towards COVID-19

4.7. The solid waste is classified according to their characteristics: as sharp, infectious and general wastes. A) Yes B) no

4.8. All staff responsible for cleaning has received training?

A) Yes B) Not all trained C) None trained

**Key informant interview**

1. What are the problems of water sanitation and hygiene within the institution?
2. What are the reasons for poor wash services in your healthcare facilities?

Thank you so much for joining me
