# Supplementary material for: Access to and challenges in water, sanitation, and hygiene in healthcare facilities during the early phase of the COVID-19 pandemic in Ethiopia: A mixed-methods evaluation
Source: PLoS One. 2022 May 13;17(5):e0268272. doi: 10.1371/journal.pone.0268272 (PMC9106162; doi:10.1371/journal.pone.0268272)
Supplement: S2 File — (DOCX) [file pone.0268272.s002.docx]

**Annex II Amharic version questionnaires**

መጠይቅ

የመጠየቁ ዋና አላማ በደቡብ ወሎ ዞን ባሉት የጤና ተቐማት ያለዉን የዉሃ የአካባቢንጽህና እና የጤና አጠባበቅሲሆን የጥናቱ ዉጤታማነት እርስዎ በሚሠጡት እዉነተኛ መረጃ ላይ የተመሰረተ ነዉ፡፡ለጥያቁዎቹ የሚሰጡት መልስ ለጥናቱ አላማ ብቻ የሚዉል ነዉ፡፡ስለዚህ እያንዳንዱን ጥያቄ በጥንቃቄ መልስ ያስቀምጡ፡፡ ለፈቃደኛነተዎ በጣም እናመሰጊናለን፡፡

**አጠቃላ ይመረጃ**

**ክፍል አንድ አጠቃላይ የጤና ተቋማት መረጃ**

1.1መጠይቁ የተሞላነትቀን-----------------------------

1.2 ተቐሙየሚገኝበትወረዳ----------------ከተማ--------------------

1.3. ተቋሙየተመሰረተበትጊዜ------------------------

1.4 የጤናተቋሙባለቤት ሀ/የግል ለ/የመንግስት

1.5.የጤና ተቐሙ መገኛ ሀ/ ከተማ ለ/ ገጠር

1.6.የጤና ተቐሙአይነት ሀ/ ክሊኒክ ለ/ ሆስፒታል ሐ/ጤናጣብያ

1.7የሰራተኞችብዛት ወ-------- ሴ--------- ድምር

1.8. በቀን በአማካኝ የሚያሰተናግደዉ በሽተኞች ብዛት--------------------

1.9 የዋሽ አስተባባሪ ተቋሙ ዉስጥ አለ ሀ/አለ ለ/የለም

1.10 የጤናተቋሙ የዋሽ ኮሚቴ አለ ሀ/አለ ለ/የለም

**ክፍልሁለት. ዉሃንበተመለከተ**

2.1. የተቋሙየዉሃመገኛምንድነዉ

ሀ. ከቧንቧለ/ የተጠበቀጉድገዋድዉሃ ሐ/የዝናብዉሃመ/ ያልተጠበቀጉድገዋድዉሃ ሠ/ የታነከርዉሃረ/ የገጸምድርዉሃ ሰ/የቦኖዉሃ ሸ/ የተጠበቀምንጭ

ቀ/ ያልተጠበቀ ምንጭ በ/ ምንም የዉሃአቅርቦት የለም ተ/ ሌላ ካለ ይግለጹ

2.2. ጤናተቋሙ አማራጭ የዉሃ ማከማቻ አለ; ሀ/አለ ለ/የለም

2.3. ዋናዉየዉሃመገኛቦታከግቢዉዉሲጥነዉ

ሀ/ አዎ ለ/አይደለም

2.4 ከግቢዉዉጭከሆንምንያህልይርቃል

ሀ/ከ 500 ሜ በታች ለ/ ከ500 ሜ በላይ

2.5. ዉሃለመቅዳተምንያህልጊዜይፈጃል(በአማካኝደቂቃ)

ሀ/ እስከ 5 ለ/ ከ 5-10 ሐ/ ከ 10-15 መ/ ከ 15 በላይ

2.6. አሁንየዉሃአቅርቦትአለ ሀ/ አለ ለ/ የለም

2.7. ባለፉትሁለትሳምነታትዉስጥሙሉቀንየመጠጥዉሃአቅርቦትነበር

ሀ/ አለ ለ/የለም

2.8. አመቱንበተመለከተየዉሃአቅርቦት

ሀ/ አዎአመቱንበሙሉአለለ/ በአብዛኛዉአለሐ/በአብዛኛዉየለም

2.9 የዉሃዉ አይነት ቧንቧ ከሆነ ስንት ቧንቧዎች አሉ------------

2.10 አገልግሎት የሚሰጡ ስንት ቧንቧዎች አሉ

**ክፍልሶስት. የአካባቢንፅህናንበተመለከተ**

3.1. በጤና ተቋሙ ዉስጥ ሽንት ቤት አለ ሀ/አዎ ለ/የለም

3.2 መልሱ አዎ ከሆነ ፣በጤናተቁሙ ዉስጥ ያለዉ የሽንት ቤት አይነት ምንድን ነዉ

ሀ/ በዉሃየሚሰራ ሐ/ ርብራብየሌለዉጉድጓድ

ለ/ ባለርብራብየጉድጓድመመናፈሻያለዉጉድጓድ ሠ / ሌላካለይጥቀሱ

3.3ጤናተቁሙስንትየሚሰራሽንትቤትአለዉ; -----------------

3.4 መጸዳጃ ቤት በር እና ቁልፍ ያለዉ ምንያህልነዉ----------------------

3.5. መጸዳጃ ቤቱ ለታካሚዎችና ለህክምና ባለሙያዎች የተለያየ ነዉ ሀ/ አዎ ለ/የለም

3.6. መጸዳጃ ቤቱ ለወነድና ሴት የተለያየ ሽንት ቤት አለዉ ሀ/ አዎ ለ/የለም

3.7 ሽንት ቤቱ በስንት ጊዜ ይጸዳል

ሀ/ በቀን ሁለት ጊዜ ሐ/ በሳምንት አንድ ጊዜ ሠ/ሌላ ካለ ይግለጹ

ለ/ በቀን አንድ ጊዜ መ/ በማነኛዉም ጊዜ

3.8 የተቋሙየሽንትቤትንጽህናምንይመስላል

ሀ/ ንጹህ ነዉ ለ/ መካከለኛ ሐ/ዝቅተኛ

3.9 መንቀሳቀስለማይችሉሰዎችየሚስማማሽንትቤትአለ ሀ/አለ ለ/የለም

3.10.መጸዳጃ ቤቱ ሲሞላ የሚወሰደዉ መፍትሄ ምንድነዉ

ሀ/ይመጠጣል ለ/ይደፈናል ሐ/ ምንም አይደረግም መ/ ሌላካለይግለጡ

**4. ንፅህና**

4.1 በተቋሙ የሚሰራ የእጅ መታጠቢያ ገንዳዎች አለ ሀ) አዎ ለ) የለም

4.2 በውሃ ብቻ ያላቸዉ የመፀዳጃ ቤቶች ብዛት---------------------------

4.3 የውሃ እና የሳሙና ያለዉ የመፀዳጃ ቤቶች ብዛት------------------------

4.4 በታኪሚዎች እንክብካቤ መስጫ ቦታዎች ላይ የእጅ መታጠቢያ አለ? ሀ/ አዎ ለ/ የለም

4.5 በእንክብካቤቦታ እና በመጸዳጃ ቤት ላይ የእጅ መታጠቢያ (ሳሙና እና ውሃ ሳኒታይዘር) አለ?

ሀ/ አዎ ለ/ የለም

4.6 በተቋሙ ዉስጥ በተለያዩ ቦታዎች ላይ የእጅ መታጠብ ፖስተር አለ? ሀ/ አዎ ለ/ የለም

4.7 ደረቅ ቆሻሻው እንደ ባህሪያቸው ይከፋፈላል ስለታም፣ ተላላፊ እና አጠቃላይ ቆሻሻዎች ተብለዉ ይከፈላሉ. ሀ) አዎ ለ) የለም

4.8 የጽዳት ኃላፊነት ያለባቸው ሁሉም ሠራተኞች ሥልጠና አግኝተዋል?

ሀ) አዎ ለ) በከፊል ሰልጥነዋል ሐ) የሰለጠነ የለም።

**ቁልፍ መረጃ ሰጭ ቃለ መጠይቅ**

1. በተቋሙ ውስጥ የውሃ አቅርበት የግልና የአካባቢ ንፅህና ችግሮች ምን ምን ናቸው?
2. በተቋሙዉስጥያለዉየዉሃ፣የአካባቢንጽህናጤናአጠባበቅም ችግር መሰረታዊ ምክንያቶቸ ምነድናቸዉ ንይመስላል

ስለተባበሩኝ በጣም አመሰግናለሁ፡፡
