# Supplementary material for: Access to and challenges in water, sanitation, and hygiene in healthcare facilities during the early phase of the COVID-19 pandemic in Ethiopia: A mixed-methods evaluation
Source: PLoS One. 2022 May 13;17(5):e0268272. doi: 10.1371/journal.pone.0268272 (PMC9106162; doi:10.1371/journal.pone.0268272)
Supplement: Attachment — Submitted filename: comment.docx [file pone.0268272.s004.docx]

**Comments**

**Abstract**

Sampling why not you use proportional allocation to the number of health care facilities since all health facilities are known by the regional health bureau.

**Line 33** add the word “by” before the word “using “ and add “interviewer administered” before “structured questioner”

**Line 50** who are nongovernmental health facilities? Change the word either NGO or partners

**Background**

**line 98** it doesn’t state in which country specifically that CoVID-19 is occurred on March 13, Hence re-write this sentence.

**Line99-100** the sentence is not complete.

**Line 101** 278 HCFs are in nationwide, or regionally or zonal level???????

**Study design**

Remove line 113,114,115 which talks about the boarder of Wello zone that much is not necessary

**Source and study population**

**Line 121** All HCFs which is present…… change the word present by the word “exist”

**Sample size determination and sampling procedures**

**Line 125** why not you mention the number of the total health facilities found in the zone to see whether the sample (70) is representative or not? And what was your base to take 70? Why not 80, 90,100 or above?

Out of the total sample, 80% are private clinics what was your base to take larger sample from private clinics.

**Line 125** Since there is enough data on the number of health institutions found in the zonal health department, why don’t you use proportional sampling technique rather than simple random to make it more uniform and representative of each health facility category?

**Line 127-131** how qualitative data were collected? Is it in-depth interview or FGDs? Explain well which method is best in exploring the challenges of an issue?

**Line 150** what about the definition of limited access for sanitation??

Data collection tools and quality assurance

**Line 165** contextualized to the **study……**

**Line 170** you use spot observation to collect data but you didn’t mention the result that you obtain through this method in your result part.

**Line 188**  how the qualitative data were analyzed? It is not exhaustively written. Hence you should mention the method that used to analyze it.

**Line 204-206**; do you think that your result represent the HCFs that are found in the zone?

**Line 206** are an environmental health professionals the only professionals who coordinates the WaSH activities? Did you ask the presence of even other professionals in the position?

**Line 207** average is not the appropriate measures rather you use mean +/- SD since the average is 55 but the highest value is 320, do you see the gap?

**Line 208**; though the highest number of patient flow is in the hospital, you included in your study only 7% of them. Do you think your sample is representative?

**Line 234** I think it is better to write “…HCFs was **unbalanced** patient load **with** functional…. ”

**Line 240** it is better to change the word “the floor” by the word “the slab”…..disposal of “faces” by “**faeces** “ after that add the word “**and”** before urine

**Line 242** ….challenges of **hygienic facilities**

**Line 243** since it is 35 or half of your sample, you can’t say more than half of…..

**Line 248** “**proper waste management**” what does it mean in this research context. It needs operational definition for this research

**Line 248** …..and they disposed **off ….** Add one “f”

**Line 249-252**; the sentence is not clear. Is it about cleaning or cleaning protocol or the janitors training status?

**Line 253-255** I didn’t understand about your sample the person from private health care facilities customer? If it is so remove it since they are two independent setting i.e governmental and private hospitals you compared two different institutions. Besides, in your sampling section, there is no list of participant from private health institution users. From where did you bring this information?

**Line 262-263** it is better to add references to the written sentence

**Line 265-267** the sentence which describes the aim should be omitted since it is already mentioned earlier

**Line 267**. Can we say all HCFs have accessed to water supply? In you result part line 214, 11% of them have no water during data collection time. Some time it is more than that. Hence you should modify this statement

**Line 267-271** you should treat each component independently otherwise there will be writing the result repeatedly which will be boring to the reader. Hence write the discussion separately for water, sanitation and hygiene

**Line 273 and 274** it needs re-writing and is it possible to put the different country status by average number? Why not you mention for each country status to know and compare it

**Line 275**; your justification doesn’t convince the reader i.e why don’t you find the similar setting to compare it either rural or urban?

**Line 278** your justification is the variation in the HCFs why not you compare with similar setting?

**Line 278** remove the bracket at the end of line 278

**Line 279-282** “almost all …….in private clinics” this statement should be placed after discussing water, sanitation and hygiene results and even compare the result and the situation with studies done in other parts of Ethiopia.

**Line 287** you said that the ratio 11.5 is greater than WHO standard. What is the WHO standard put the number. Besides, there is no reference, hence put your reference

**Line 288** the statement “….between the level….” Should be changed to between the HCFs. Even it is better to put the ratio result (in number) with in each HCF.

**Line 291-301** the paragraph seems the result part since you didn’t discuss any of your result by comparing your result with the other studies. Hence this paragraph needs re-writing again

**Line 304** “HCFs had no function…..” change the word function to “functional”

**Line 305** what about the other study setting you didn’t discuss your finding simply put the number that you wrote in the result part here again.

**Line 307-309**; this justification is for the 60% (HCFS had no function hand washing facilities) or 21% (HCFs had functional handwashing facilities with water and soaps)? It is confusing discussion part. Hence it is better to treat independently.

**Line 314-320** you simply put your result again that you wrote in your result part. Hence it needs further discussion by comparing your result with other findings and give your justification why the variation occurs.

Where is the qualitative data discussion part? you didn’t put anything. Why you collect the qualitative data? And how you analyzed it? Is it by open code, thematic, ATLAS-Ti or by what technique? Nothing is said about this. Please write something on it. You said in your methodology mixed where is the mixed nature? I didn’t see it or realized it.

**Line 323** “exclusion of health post” I preferred you were included these health post rather than the private clinics that you included. The reason is that

1. There is a mix up of different setting that is governmental and private which are almost completely different setting in Ethiopian condition
2. Your study design is not comparative cross sectional method
3. You didn’t compare the result of private clinics with the government or even to the other similar setting of different countries. The data that you got from private clinics are confined in the governmental HCFs and treated as they are governmental HCFs

**Limitation** part what about the cross-sectional nature of the data?

**Line 335** make correct on the word “…..concerned exerts……” change to **concerned experts**

**Annex**

**Table 3 on page 24** “overall latrine cleanliness” i.e high, medium, low, it needs operational definition.
