# Supplementary material for: Access to and challenges in water, sanitation, and hygiene in healthcare facilities during the early phase of the COVID-19 pandemic in Ethiopia: A mixed-methods evaluation
Source: PLoS One. 2022 May 13;17(5):e0268272. doi: 10.1371/journal.pone.0268272 (PMC9106162; doi:10.1371/journal.pone.0268272)
Supplement: Attachment — Submitted filename: response to the reviwers.docx [file pone.0268272.s006.docx]

**Response to Reviewer 1**

#1:  I continue to be concerned with the wider use of literature. For the use of “Worldometer” references 18 and 22, I would prefer to see official Ethiopian government sources. Also, the use of a WHO/UNICEF 2015 JMP report in reference 38. Please update and use the most recent 2021 WHO/UNICEF report.

**Response:-thank you very much for your comments. Hence, we have incorporated your comments in the revised version of the manuscript.**

2.) The study presents the results of original research, but does not compare to other recent studies in Ethiopia or other low- and middle-income countries during COVID-19.

**Response:-thank you for your constrictive comment too. We have tried to use researches which were conducted in Ethiopia and other low and middle countries as much as possible in the revised manuscript.**
3.) As previously commented, the authors have not made this change. “The N=70, there is not a large enough dataset to have results to 0.1%. Please round all results presented to the nearest whole number.” In Revision 1, Table 1 and 2 still list results to 0.1%

**Response:-ok we have modified it based on your comments in the revised manuscript (see the revised version).**
4.) Finally, when you submit the corrected version, please do check thoroughly, in order to avoid grammar, syntax or structure/presentation flaws - please seek for professional English proofreading services or ask a native English-speaking colleague of yours in order to refine and improve the English in your paper.

**Response:-thank you for the comment; we have tried to incorporate all issues you have raised.(see the revised version of the manuscript)**

**Response to reviewer 2**

#1: all comments that I raised in the first round were properly addressed by the authors. That was nice. But I have one comment on the reference section. Your references are not similar in style. Hence you should rewrite all references again

**Response:-thank you for the comment. We have modified the references based on the standard guideline of the journal. (see the revised version of the manuscript)**
