# Supplementary material for: Access to and challenges in water, sanitation, and hygiene in healthcare facilities during the early phase of the COVID-19 pandemic in Ethiopia: A mixed-methods evaluation
Source: PLoS One. 2022 May 13;17(5):e0268272. doi: 10.1371/journal.pone.0268272 (PMC9106162; doi:10.1371/journal.pone.0268272)
Supplement: Attachment — Submitted filename: response to reviewers.docx [file pone.0268272.s007.docx]

**Question #1** I have assessed your submission, and whilst the science is now adequate, I have concerns about the overall readability of the manuscript. I therefore request that you revise the text to fix the grammatical errors and improve the overall readability of the text.

**Response:** **Thank you for this remark. We have revised the whole manuscript to reduce the grammatical errors and improve the overall readability of the text by the help of known researcher Dr Metadel Adane Mesifin (Please see the revised version for each section).**

**Question #2** Please review your reference list to ensure that it is complete and correct. If you have cited papers that have been retracted, please include the rationale for doing so in the manuscript text, or remove these references and replace them with relevant current references. Any changes to the reference list should be mentioned in the rebuttal letter that accompanies your revised manuscript. If you need to cite a retracted article, indicate the article’s retracted status in the References list and also include a citation and full reference for the retraction notice.

**Response: thank you very much for critical comment. Hence we have improved the whole listed references using the guideline of PLOSE ONE (see the revised version for each section).**
